# Living Kidney Donation: The Outcomes for Donors

**Published:** 2010-05-01

**Authors:** A. J. Ghods

**Affiliations:** *Division of Nephrology and Transplantation Unit, Hashemi Nejad Kidney Hospital, Iran University of Medical Sciences, Tehran, Iran*

**Keywords:** Kidney transplant, Kidney donor, Living donor, Donor outcome

## Abstract

During the past decade, the number of transplantation from living kidney donors has substantially increased worldwide. The rate of increase varies from one country to another. The risk of unilateral nephrectomy to the donor includes perioperative mortality and morbidity plus the long-term risk of living with a single kidney. The rate of perioperative mortality and morbidity is about 0.03% and 10%, respectively. More attention is required to prevent serious complications of laparoscopic donor nephrectomy. A grading system in recording perioperative complications is necessary for making it available to each potential donor. The number of studies on long-term outcome of living donors is very limited. The overall evidence suggests that the risk of end-stage kidney disease is not increased in donors, however, mild renal failure, hypertension and proteinuria are not uncommon in living donors. There is also concern that the incidence of cardiovascular disease may be higher in kidney donors. Establishing living donor registry and follow-up is extremely important. Only through these registries the long-term risk of kidney donation will become more apparent. Because of severe shortage of transplantable kidneys, some transplant centers are now using donors with comorbidities and few centers are involved in transplant tourism with inadequate donor screening and follow-up. Prevention of these unacceptable practices in living kidney donors was emphasized in Amsterdam Forum in 2004 and Istanbul Summit in 2008.

## INTRODUCTION

The first successful living donor kidney transplant was performed between identical twins by Dr. Joseph Murray at the Peter Bent Brigham Hospital in Boston, USA, in 1954. Now, after over half a century, living donor kidney transplantation has become the treatment of choice for the majority of patients with end-stage kidney disease. Only in 2006, about 27,000 kidney transplants from living donors were performed worldwide; 1615 (0.6%) of these transplants were carried out in Iran. Because of his prominent contribution to the advances in medicine, Dr. Joseph Murray was awarded the Nobel Prize in December 10, 1990. 

The living kidney donation for transplantation is unique among major surgical operations in that a perfectly healthy person is exposed to the risk of surgery not for his own benefit but entirely for the benefit of another individual. The risk of unilateral nephrectomy to the donor includes perioperative or short-term mortality and morbidity, plus the long-term risk of living with a single kidney. 

In this article; first, I will very briefly, review the fact that during the last decades the number of living kidney donations for transplantation has substantially increased worldwide. In the second part, I will discuss the short-term complications in living kidney donors reviewing the perioperative donor mortality and morbidity. In the third section, I will review several studies on long-term outcome of living kidney donors with emphasize on the impact of unilateral nephrectomy on the development of proteinuria, hypertension, reduced renal function and end-stage kidney disease. Another issue is the long-term risk of developing cardiovascular disease in living kidney donors. Because lower glomerular filtration rate (GFR), proteinuria and hypertension that are known risk factors of cardiovascular disease are not uncommon in living kidney donors, especially in long-term. Finally, I will briefly discuss two other relevant topics: The need for living kidney donor registry for long-term follow-up and the impact of severe shortage of transplantable kidneys on the outcome of living kidney donors.

**I- THE NUMBER OF TRANSPLANTATION FROM LIVING KIDNEY DONORS CONTINUES TO INCREASE WORLDWIDE**

During the last decades, the number of living kidney donations for transplantation in almost all regions of the world has substantially increased and it is expected that it will continue to rise in upcoming decades. The main reason is that, as the prevalence of end-stage renal disease steadily rises in each country, the rate of living kidney donation in the world continuously increases. In a recent study on “global trends in the rate of living kidney donation,” Horvat and coauthors showed that over the last decade, the number of living kidney donor transplants have grown at least 50%.[1] According to that study, the greatest number of living donor kidney transplants on a yearly bases (2006) have been performed 6435 in the United States, 3207 in India, 1768 in Brazil, 1615 in Iran, 1459 in Mexico, 1186 in Pakistan and 939 in Japan. These are the crude number of living kidney donor transplants. The authors provided data in the form of rate per million population (pmp) to allow for a better comparison of living kidney donor transplantation activity among countries. In those data, Saudi Arabia had the highest living kidney donor transplant rate of 32 pmp followed by Jordan (29 pmp), Iceland (26 pmp), Iran (23 pmp) and the United States (21 pmp). The data of Saudi Arabia should be interpreted carefully because only one-third (10 pmp) of all these living donor kidney transplants had been performed inside Saudi Arabia and the remaining two-thirds were carried out abroad by transplant tourism. Iran was ranked fourth worldwide because it is the only country in the world that has adopted a paid and regulated living unrelated kidney donation program. This ethically controversial system, known as the Iranian model, has eliminated the waiting list for kidneys. About 75% of all kidney transplants in Iran are being performed by paid volunteering kidney donors.[2] 

The current proportion of all kidney transplants from living and deceased donors shows a significant geographical difference in living donor transplant activity among various countries. In most European countries such as Poland, Spain, Italy, France and Germany the rate of living kidney donor transplantation is small (2%–18%), because the majority of renal transplantations in these countries are being performed from deceased donor sources. In some other countries such as Sweden, Switzerland, United Kingdom, United States and Canada in addition to having a large deceased donor transplantation program, increasing number of renal transplantation from living donors are also being performed (26%–42%). In the third group of countries such as Brazil, Korea, Turkey, Japan and Iran the majority of kidney transplants are from living donors (55%–80%). This is because of cultural problems as in the case of Japan, and because of cultural problems plus infrastructural deficiencies as in Brazil, Turkey and Iran. Finally, in some other countries such as Egypt, Jordan, Pakistan, Oman and Iceland there is no deceased donor transplant program and all (100%) of kidney transplants are carried out from living donors.[1]

Currently, more patients with end-stage kidney disease prefer to receive a renal transplant than to remain on dialysis, because since long time ago, it has been shown that a renal transplant significantly prolongs survival and provides better quality of life compared with dialysis.[3,4] But those patients opting for a renal transplant must decide whether to have a living donor transplant or to register to the waiting list for a deceased donor transplantation.

Because of the following reasons, the majority of patients with end-stage renal disease prefer to receive living donor transplants from either a related or an unrelated donor. It has been documented that recipients of living donor kidneys have a better graft survival than recipients of deceased donor kidneys. This is true for living related donor and also for living unrelated donor transplantation. In addition, it has been shown that the outcome of living unrelated donor renal transplants is superior to deceased donor renal transplants and similar to one haplomatch living related donor transplants. This is one of the main reasons why the number of living unrelated donor transplantations are steadily increasing around the world.[5] Another reason is the recognition that the outcome of renal transplantation is inversely related to the duration of pretransplant dialysis.[6] In other words, the shorter the period on dialysis the better graft survival. As a result, many patients are willing to undergo living donor kidney transplant sooner in the course of their disease as preemptive transplantation with expected superior outcome rather than waiting to receive a deceased donor transplantation. The other cause of increasing number of living kidney donations for transplantation is the introduction of laparoscopic donor nephrectomy. This procedure is associated with less pain and faster recovery of donor compared with the conventional open donor nephrectomy. As a result of this attractive approach, more and more potential living donors accept kidney donation. 

A very important piece of information that became very useful for members of transplant community and transplant policy makers is the findings came from the study of Sheehy and coworkers. In that study, authors predicted the annual number of brain-dead potential donors in the United States and showed that even if the organs of all brain-dead potential donors could have been procured the supply of kidneys would still be inadequate to meet the escalating demand of patients in need. In other words, the number of kidneys available from deceased donor sources will never be enough and living kidney donation for transplantation is needed to comply with the demand.[7]

**II- SHORT-TERM COMPLICATIONS OF LIVING KIDNEY DONATION**

In this section, perioperative donor mortality and morbidity will be reviewed. There are also two related issues to be mentioned. The first is about the laparoscopic donor nephrectomy technique. This less invasive approach has increased the number of living kidney donations. However, since introduction of laparoscopic donor nephrectomy, questions have been raised on the impact of this procedure on the rate of perioperative complications and whether it has increased donor mortality and morbidity rates compared with open donor nephrectomy? Because there is no consensus agreement and no clear answer to this question, several reports that compare these two kidney donation techniques will be reviewed. Another issue is the lack of a grading system or standardized classification for reporting perioperative complications. Current reporting system makes comparison of the complication rates among transplant centers difficult and most importantly, makes obtaining an ethically acceptable informed consent from living donors unsatisfactory. According to ethical principles, all relevant information about kidney donation should be clearly disclosed so that any potential donor can understand all the information that has been disclosed. So a grading system that has been proposed by some authors to eliminate this problem will be mentioned.[8]

**PERIOPERATIVE LIVING KIDNEY DONOR MORTALITY**

It is obvious that the death of a living kidney donor is a terrible disaster for members of every transplant center and most transplant teams are not willing to report the death of their living donors. This is why obtaining a reliable data on donor death is not easy and it should be done by a kind of study that surveys a number of transplant centers but keeps the name of centers confidential. The first of such studies was published by Najarian and his group in 1992. To asses the perioperative mortality in living kidney donors, the authors surveyed all members of the American Society of Transplant Surgeons about donor death at their institutions. They documented 17 perioperative donor deaths in the United States and Canada and estimated the living kidney donor mortality to be 0.03%.[9] To find out the rate of donor mortality in Iran, we surveyed at least one staff from each transplant center about donor death. By the end of 2004 that over 18,000 live donor nephrectomies had been performed throughout the country we documented four perioperative donor deaths, making donor mortality rate 0.02%.[10] Unfortunately, one of these four donor deaths happened in our transplant center. The donor died of severe internal bleeding within 24 hours after nephrectomy. In our transplant center, over 2500 live donor kidney transplantation have been performed. Donor mortality rate of our center was calculated to be 0.04%. In one comprehensive study, Matas and coworkers surveyed 234 UNOS listed transplant programs in the United States to determine the living kidney donor mortality and morbidity for open and laparoscopic donor nephrectomy. The 171 respondent transplant centers had performed 10,828 live donor nephrectomies. In this survey, two donors had died of surgical complications (donor death rate of 0.02%). One donor was also in a persistent vegetative state. Both donor deaths and development of persistent vegetative state in another donor all were complications of laparoscopic donor nephrectomy.[11]

**PERIOPERATIVE LIVING KIDNEY DONOR MORBIDITY**

Many transplant centers have published their experience on perioperative complications in living kidney donors. In 2004, we reported perioperative donor morbidity in 1625 open donor nephrectomies performed in Hashemi Nejad Kidney Hospital, Tehran, Iran. There was a total of 162 (10%) perioperative complications that we arbitrarily divided into 24 (1.5%) major and 138 (8.5%) minor complications. Reoperation was necessary in 15 donors for bleeding in four, retained drain in two and infectious complications in five. In 22 donors readmission was required; two of them were reoperated—one for a retained surgical forceps and another for hernia. The remaining 20 were treated medically and recovered. The rate of major and minor complications in our center was very similar to the perioperative complication rates reported by other conventional transplant centers. 

Open donor nephrectomy is very safe, both for the donor and for the kidney, however, it is associated with pain and increased risk of wound-related complications. These disincentives prompted surgeons to the advent of laparoscopic donor nephrectomy that was used for the first time in 1996.[12] Since then, this approach rapidly gained popularity. Currently, the majority of transplant centers worldwide are performing living donor nephrectomies laparoscopically. But laparoscopic donor nephrectomy also is associated with surgical risks of injury to the renal pedicle resulting in internal hemorrhage. So concerns still exist as to the safety and efficacy of the laparoscopic approach. Tooher, *et al*, performed a systematic review of laparoscopic live donor nephrectomy comparing with open donor nephrectomy.[13] In this meta-analysis, 44 published studies were included. In terms of safety for donors, there did not seem to be any significant difference between open and laparoscopic approaches. The complication rates were similar; although the types of complications differed between the two donation techniques. Donor postoperative recovery appeared too superior for laparoscopic donor nephrectomy, making it more attractive for living donors. A large single center experience with laparoscopic donor nephrectomy also was reported by Jacobs, *et al*, who described their six-year experience with 738 consecutive laparoscopic donor nephrectomies from the University of Maryland.[14] Major intraoperative complications occurred in 6.8% and major postoperative complications occurred in 17.1% of cases. Conversion to open nephrectomy was in 1.6% of cases and blood transfusion was required in 1.2%. They concluded that the risks of laparoscopic donor nephrectomy to the donor must not be minimized. However, although the surgical technique and complication management have improved, it still requires an intense level of attention to prevent complications. Simforoosh, *et al*, from Iran conducted a comparative trial between 100 open donor nephrectomy and 100 laparoscopic donor nephrectomy.[15] They reported perioperative complication rate of 27% in open and 21% in laparoscopic donor nephrectomy cases. Authors concluded that compared to open donor nephrectomy, laparoscopic approach was associated with greater donor satisfaction and less morbidity. As mentioned earlier, Matas, *et al*, surveyed 234 UNOS-listed transplant programs to determine living donor morbidity and mortality for open nephrectomy, hand-assisted laparoscopic nephrectomy and non-hand-assisted laparoscopic nephrectomy. Of the 234 centers, 171 (73%) responded that had performed 10,828 living donor nephrectomies between 1999 and 2001—52.3% open, 20.7% hand-assisted and 27% non-hand-assisted laparoscopic donor nephrectomies. Morbidity after each donation technique was assessed by the rate of readmissions, complications and complications requiring reoperation.[11] In this survey, they found readmission and reoperation rates all higher for laparoscopic donor nephrectomy compared with open donor nephrectomy (P=0.001). Although the authors concluded that their survey provide current data from which comprehensive informed consent can be obtained from donors, others emphasize that establishing a grading system or standardized classification for these potential complications is necessary for presenting more understandable data to potential living donors.

Clavien, *et al*, were first to propose a classification to stratify negative outcomes in surgery.[16] Subsequently, their grading system has been modified to be applicable to outcomes in liver transplant recipients.[17] Kocak, *et al*, have modified Clavien classification and have proposed a grading system for complications of living kidney donation. Complications are graded in four groups as in the original classification. Grade one complications include all events which if left untreated have a spontaneous resolution or need a single bedside procedure. Grade two complications differ from grade one in that they are potentially life-threatening and usually require some form of intervention. Grade three complications produce lasting or residual disability and the grade four events are resulting in renal failure or death. The development of a grading system is an essential step in recording perioperative complications in a better and understandable way and in making it available to each potential donor as part of donors’ informed choice process.[8] 

**III- LONG-TERM OUTCOME OF LIVING KIDNEY DONORS**

In contrast to the large number of papers published on perioperative complications, the number of studies reporting on long-term outcome of living kidney donors is very limited. Considering the life expectancy, the long-term outcome of living kidney donors should determine the outcome of donors at least after 20 to 30 years of kidney donation. The main concern is to see whether kidney donation has any long-term impact on survival or on development of renal and cardiovascular disease.[18] 

**IMPACT OF KIDNEY DONATION ON LONG-TERM DONOR SURVIVAL**

Fehrman-Ekholm, *et al*, from Sweden conducted a study almost 10 years ago and found that kidney donors live longer.[19] By using national registry, they traced all 430 living donors residing in Sweden. All nephrectomies had been performed in one center from 1964 until the end of 1994. Donor survival was analyzed using the Kaplan-Meier survival analysis. The expected survival was computed using national mortality data. After 20 years of follow-up, 85% of the donors were alive, whereas the expected survival rate was 66%. Survival was much better in donor group. The better survival rates of kidney donors in this study does not mean that kidney donation *per se* increases life expectancy, but rather it reflects the careful selection of healthy individuals suitable for kidney donation and this data contradict the concept that donor longevity may be decreased by kidney donation. Twenty years after kidney donation, one-third of the donors had hypertension. Proteinuria was found in 11 donors; only four had a protein excretion of more than 1 g/24 hrs. There was a deterioration in the renal function with increasing age, similar to what is seen among healthy subjects.

Ibrahim, *et al*, studied donor survival in 3,698 kidney donors who donated kidneys during the period from 1963 through 2007.[20] They showed that the survival of kidney donors was similar to that of controls selected from the general population who were matched for age, sex, race or ethnicity. This study, however had a selection bias since all kidney donors were carefully screened for disease, whereas the control subjects selected from the general population were not screened.

**IMPACT OF KIDNEY DONATION ON DEVELOPMENT OF RENAL DISEASE IN LONG-TERM**

Reduction in GFR, development of hypertension and/or proteinuria are not uncommon in living kidney donors. However, the overall evidence suggests that the risk of end-stage kidney disease is not increased. Ibrahim, et al, studied a subgroup of 255 living kidney donors at a mean±SD of 12.2±9.2 yrs after kidney donation. They showed that 14.5% of donors had a GFR lower than 60 mL/min/1.73 m^2^ body surface area; 32.1% had hypertension and 12.7% had albuminuria. Older age and higher body mass index, but not a longer time since donation, were associated with both a GFR less than 60 mL/min /1.73 m^2^ body surface area and hypertension. A longer time since donation, however, was independently associated with albuminuria. Female sex was a risk factor for a low GFR—surprisingly, smoking status was not. Albuminuria was also less likely to develop in woman.[20] End-stage renal disease that necessitated dialysis or transplantation developed in 11 of 3,698 donors 22.5±10.4 years after donation. Of 11 donors who developed end-stage kidney disease seven were women and eight were white. The estimated incidence of end-stage renal disease in donors appeared to be 180 per million persons per year which is comparable to the overall adjusted incidence rate of 268 per million person per year in the white population of the United States. The authors concluded that according to their study, kidney donors have a normal life span, and a health status that is similar to that of the general population, and that donors do not have an excessive risk of developing end-stage kidney disease in long-term. Authors also showed that the majority of kidney donors had a preserved GFR in long-term, and that their rate of albuminuria and hypertension were similar to those of matched controls.[20] But this study has certain limitations: First, kidney donors who were carefully screened for disease were compared with controls from the general population who were not screened. Davis, *et al*, have argued that the good survival and lower rate of end-stage renal disease in this study may have been due to the optimal condition of the donors at the time of donation. According to them, the ideal control group would not be selected from the general population; they should be selected from a group of people living in the same area who have been evaluated as candidates for donation but have not donated.[21] Secondly, because the majority of kidney donors were white, the finding of this study cannot be taken as the final word for all races. Potential donors of nonwhite ethnic groups such as Afro-Americans need to be very carefully screened and counseled for kidney donation.

Gibney, *et al*, found an indirect way of studying the question of excess risk in Afro-American kidney donors.[22] They used the United Network for Organ Sharing/Organ Procurement Transplantation Network (UNOS/OPTN) database and searched for patients who previously donated a kidney and were subsequently placed on the kidney transplant waiting list. Then, they compared the race of donors listed for kidney transplant to the race of all living donors during the same time period. Although Afro-Americans comprised 14.3% of all living kidney donors, they constituted 44% of donors reaching the waiting list (P<0.001). These data provided indirect evidence that the risk of renal failure may be higher in Afro-American donors.

**IMPACT OF KIDNEY DONATION ON DEVELOPMENT OF CARDIOVASCULAR DISEASE IN LONG-TERM**

Currently, long-term outcome of living kidney donors is only evaluated by renal morbidity and mortality measured by factors such as reduced renal function (low GFR), occurrence of proteinuria, hypertension, and development of end-stage renal disease and even death. During the last decade, a number of papers have been published indicating that elevated serum creatinine (low GFR) and proteinuria are independent predictors of cardiovascular mortality.[23-25] Because mild renal failure, proteinuria and hypertension which are risk factors for cardiovascular disease are not uncommon in living kidney donors; there is concern that the incidence of cardiovascular disease may be higher in kidney donors. As a result, there is a suggestion that some well-designed studies need to be conducted to see if kidney donation *per se* predisposes some donors to cardiovascular disease. To conduct this study a large pool of data (registry) from living donors is needed.

**IV- THE NEED FOR ESTABLISHING LIVING KIDNEY DONOR REGISTRY AND LONG-TERM FOLLOW-UP**

Many experts believe that living donors should be offered life-long medical follow-up. This regular long-term follow-up will be for the benefit of donors because it identifies medical conditions that are risk factors for donors’ health such as hypertension, proteinuria and reduced GFR which are not uncommon in kidney donors. Currently, because of severe shortage of transplantable kidneys, some transplant centers are accepting donors who are older, who are obese, who have hypertension or who have other comorbidities. Regular follow-up of these donors is extremely important. Another benefit of long-term follow-up of living donors is reporting their data to a national or supranational registry. Only through establishing kidney donor registries the long-term risk of kidney donation will become more apparent. The value of living kidney donor registries will be similar to the major kidney recipient registries as UNOS/OPTN, CTS, SRTR and ANZDATA.[26] Currently, there are some national living kidney donor registries in Switzerland, Sweden and the United Kingdom. Since 1999, UNOS has required transplant programs to report information about living kidney donors at postoperative discharge, six months, and 12 months. In 2007, this reporting requirement has been extended to 24 months. As can be seen, US transplant centers are required to report follow-up of living donors only for two years post-donation. In a recent study, Mandelbrot, *et al*, clearly showed that this follow-up and reporting is also associated with some barriers.[27] In that study, the authors surveyed US transplant centers to asses practices and barriers in providing follow-up care to living donors. They showed that the adherence of donors to recommendation of programs for donor follow-up decreases sharply from 91% in six weeks to 7% in five years post-donation. The main barrier to collecting more complete information were donor inconvenience (84%), and lack of reimbursements to the transplant center for providing follow-up care (50%). This services were paid mainly by donors—58% by donors health insurance and 25% donors paid out of pocket. They concluded that significant changes are required to improve long-term follow-up of living donors by US transplant centers. 

**V- IMPACT OF SEVERE SHORTAGE OF TRANSPLANTABLE KIDNEYS ON THE OUTCOME OF LIVING KIDNEY DONORS**

Severe shortage of transplantable organs including kidneys is the most serious problem in transplantation medicine today. Shortage of transplantable kidneys is associated with several undesirable consequences. As an example, many patients are dying each year while waiting for a deceased-donor kidney. But two of several consequences of kidney shortage have some negative impact on the outcome of living kidney donors. The first problem is that currently some transplant centers are accepting kidney donors with comorbidities. It is obvious that kidney donation by a donor who is old or obese or have mild hypertension or mild diabetes may result in serious medical conditions in kidney donor in the future. The second unacceptable consequence of severe shortage of transplantable kidneys is the increasing number of commercial transplants and transplant tourism. According to World Health Organization (WHO), about 10% of all kidney transplants performed around the world are commercial transplants.[28] In commercial transplantations and transplant tourism, the main purpose is to restore a healthy life for the recipient. Unfortunately donors are not carefully screened for disease before kidney donation and have no appropriate follow-up after the surgery. As a result, the rates of post-kidney-donation morbidities are higher in this group of donors. 

To eliminate many problems present in living kidney donation including these two unacceptable consequences of severe kidney shortage, two international meetings were organized by Dr. Francis Delmonico, Director of Medical Affairs of The Transplantation Society. The first meeting was held in Amsterdam in April 2004, focused on the care of living kidney donors; the second meeting was held in Istanbul in May 2008, focused on organ trafficking and transplant tourism. 

In Amsterdam Forum, over 100 experts and leaders in transplantation, representing over 40 countries across the world participated. One of the Consensus Statements of this Forum was that “Because of the need for more transplantable kidneys, persons with conditions that may increase the health risks for the donor are currently being considered and used as donors. The international transplant community recommends that acceptance of such individuals as kidney donors be conducted in an ethical manner, accounting for the autonomy and safety of the donor and with rigorous attention to clinical outcome.”[29] In Istanbul Summit on Organ Trafficking and Transplant Tourism, also over 150 representatives of scientific and medical bodies from both developed and developing countries attended. One of the statements of the Declaration of Istanbul is that “Organ trafficking and transplant tourism violate the principle of equity, justice and respect for human dignity and should be prohibited.”[30]
